# Recent Developments in RFT Encourage Interbehavioral Field-Based Views of Human Language and Cognition: A Preliminary Analysis

**DOI:** 10.1007/s40614-024-00407-3

**Published:** 2024-05-09

**Authors:** Colin Harte, Dermot Barnes-Holmes

**Affiliations:** 1 Departmento de Psicologia, Universidade Federal de São Carlos, São Carlos, Brazil; 2 Instituto Par—Ciências do Comportamento, São Paulo, Brazil; 3 Instituto Nacional de Ciência e Tecnologia sobre Comportamento, Cognição e Ensino, Brazil; 4 School of Psychology, Ulster University, Coleraine, Northern Ireland

**Keywords:** RFT, DAARRE, Interbehavioral field, Psychological event, IRAP

## Abstract

Relational frame theory (RFT) as a behavior-analytic approach to understanding human language and cognition is now over 40 years old. However, the last 8 years have seen a relatively intense period of empirical and conceptual developments within the theory. Some of this work has begun to draw on early and much underplayed features of RFT, including field-theoretical analyses and concepts. These analyses are relatively nascent and thus the current article aims to provide a relatively detailed example of a field-theoretical analysis of a specific RFT research program. We begin with a brief overview of the “traditional” RFT approach to human language and cognition, followed by a summary of recent research involving the implicit relational assessment procedure (IRAP) and the differential arbitrarily applicable relational responding effects (DAARRE) model. We then go on to consider the DAARRE model in the context of J. R. Kantor’s interbehavioral formula for the psychological event. Having done so, we conclude that the challenge involved in analyzing increasingly complex forms of human language and cognition appears to call for more field-based theorizing in some form or another.

So long as investigators continue to interact with their subject matter, they will move forward to fuller understanding and scientific knowledge in psychology. Passing trends and fads of equipment, or "sophisticated" methodology, of systematic viewpoint, and of theories may accelerate or slow this movement, but they will not stop it. Time, in which research (however misguided) continues, will inevitably lead us all to interbehaviorism, if not necessarily to its vocabulary.

This personal history may prove the paradigm—where time after time, when I thought I had reached a new position, I'd stop myself short . . . "Hey, wait a minute, Kantor wrote that"—or "that's what Kantor would say." He's always been there first.

This is the way it will happen for others, over coming years. (Verplanck, 1983, p. xxv)

It has been over 40 years since the first presentation of relational frame theory (RFT) as a behavior-analytic approach to understanding human language and cognition (Hayes & Brownstein, 1985), and almost a quarter of a century since the publication of the seminal volume (Hayes et al., 2001). In that time, many conceptual and empirical strides have been made. In particular, the last 8 or so years have seen a period of intense empirical and conceptual updating of the account (see Barnes-Holmes & Harte, 2022a, for a detailed description of some of these updates that are directly relevant to the core thesis of the current article). It is paradoxical, however, that these recent advances appear to be drawing on early and much underplayed features of RFT, including field theoretical analyses and concepts, which are assisting in recent RFT-based experimental analyses. Although these analyses are just beginning to evolve, we believe there is strong potential for this work to help move the study of human language and cognition within the behavioral tradition into new and exciting areas. Before elaborating on this work, however, we will begin with a brief overview of the “traditional” RFT approach to human language and cognition, which will allow us to put these recent advances in their appropriate historical context.

## The Traditional RFT Account

According to RFT, there are three fundamental properties that define a relational frame: *mutual entailment*, *combinatorial entailment*, and the *transformation of stimulus functions*. Mutual entailment refers to a bidirectional relation between two stimuli. For example, if A is taller than B, this mutually entails that B is shorter than A. Combinatorial entailment refers to novel relations that emerge between and among stimuli when three or more stimuli are related. For example, if A is taller than B and B is taller than C, then additional relations will emerge such that A is taller than C and C is shorter than A. The transformation of stimulus functions refers to a change in the functions of one or more stimuli in a frame resulting from a change in the functions of other stimuli in that frame. For example, if X, Y, and Z are coordinate, and reinforcing functions are established for X (through direct pairing), Y and Z may also acquire reinforcing properties. It is critical to note that this transformation of stimulus functions occurs in the absence of direct reinforcement.

The distinction between the transformation of stimulus functions and relational entailment is fundamental within RFT, in that together, synergistically, they provide a core unit of analysis. In making this distinction within the analytic unit, RFT assumes that these properties (entailment and transformation of functions) come under different classes of contextual control.

Specifically, Crel contextual cues control the type of relation (e.g., coordination, comparison, difference, etc.), thus determining the entailment properties, and Cfunc contextual cues control the behavioral functions produced during this relating, thus determining the transformation of function properties. For RFT, therefore, both types of contextual control are crucial in analyzing how entailment and transformations of functions combine in any given instance of [arbitrarily applicable relational responding] AARR. (Barnes-Holmes & Harte, 2022a, p. 243)

Within the RFT literature, consideration of the dynamic between entailed relations and transformations of stimulus functions has largely considered the ways in which establishing certain entailed relations allows for particular changes in the functional properties of stimuli participating in those entailed relations. As noted above, for example, if X, Y and Z participate in a frame of coordination and a reinforcing function is established for X, the function may emerge for Z. In this case, the focus is on the impact of relating on the functions of the stimuli within the relational network. In recent research, however, a greater focus has been placed on the impact of the functional properties of the stimuli on their relational properties, which we will explain below. The reader should note that some previous research has reported behavioral effects consistent with this approach (i.e., function-to-relation rather than relation-to-function), generally showing that functional classes could generate equivalence relations (e.g., Sidman et al., 1989; Smeets et al., 1997). For example, if a specific response function was established for three stimuli, and a different response function was established for another three stimuli, the two sets of stimuli may produce matching responses consistent with previously established functions. To illustrate, imagine if A1, B1, and C1 all controlled response function 1, and A2, B2, and C2 all controlled response function 2. Participants may subsequently match each of the class 1 stimuli to each other and each of the class 2 stimuli to each other in the absence of direct reinforcement. Although these findings are important, they have mostly simply shown that functional classes can generate equivalence relations. They have not, however, indicated that the functional properties of stimuli can have an impact upon the properties of relational responding itself.^1^ In order to fully appreciate the argument we are making here, we need to first describe a widely used RFT methodology known as the implicit relational assessment procedure (IRAP).

## The IRAP as an Assessment of Natural Verbal Relations

The IRAP is a computer-based task that requires participants to respond with speed and accuracy to specific stimuli deemed to be either consistent or inconsistent with participants’ preexperimentally established learning histories.^2^ On each IRAP trial, a label stimulus (e.g., “flower” or “insect”) appears at the top of the screen, and a target stimulus—such as “pleasant,” “good,” “unpleasant,” or “bad”—appears in the middle of the screen. On each trial, two response options are presented that specify particular relationships between label and target stimuli. For example, “flower” and “pleasant” might appear on a given trial with the response options “similar” and “different.” In this case, participants would be required to relate flowers as similar to, or different from, pleasant. The IRAP requires opposite patterns of responding across successive blocks of trials. For example, “flower” and “pleasant” would require the response “similar” on one block and “different” on the next. This was based on the assumption that, all things being equal, the response pattern that has been reinforced more often in the past (and is thus more probable), or one that is relationally coherent with that pattern, would be emitted more readily (Barnes-Holmes, et al., 2010). To increase the likelihood that the more probable response is observed, responding on the IRAP is typically placed under time pressure (e.g., participants are required to respond within 2000 ms on each trial).

The IRAP is usually scored by subtracting the mean response latency for one pattern of responding from the mean response latency of the opposite pattern of responding. The resulting difference, if any, is deemed to be reflective of the extent to which the patterns are consistent versus inconsistent with an individual’s verbal or relational history. In most IRAP studies, four difference scores are calculated, one for each of the four trial types typically presented within the IRAP (e.g., flowers–pleasant; flowers–unpleasant; insect–pleasant; insect–unpleasant). The “predicted” pattern of responses might thus be faster responses when confirming, rather than denying, that flowers are pleasant and insects are unpleasant; and denying, rather than confirming, that flowers are unpleasant and insects are pleasant.

Differences in the size of the trial-type effects were previously explained in terms of the differential valences of the stimuli involved (e.g., the assumption that flowers are generally positively valenced relative to negatively valenced insects). However, when nonvalenced stimuli were inserted into the IRAP in later research, specific patterns emerged that could not be readily explained in terms of differential valence. For example, differential trial-type effects were observed when participants simply had to confirm whether a color was a color or a shape was a shape (Finn et al., 2018). In particular, the difference scores for the color–color trial-type were significantly larger than for the shape–shape trial-type. This difference was unexpected because these two trial-types required the same response option within each block of IRAP trials (in this particular case it is important to note that the response option “True” rather than “False”), and did not differ in any obvious way in terms of their valence (i.e., there was no basis for a strong preference for colors over shapes). This, and similar differential trial-type effects, could not be readily accounted for by existing models of IRAP performances (Barnes-Holmes et al., 2010). As a result, a new conceptual account was proposed: the Differential Arbitrarily Applicable Relational Responding Effects (DAARRE) model (Finn et al., 2018), to which we now turn.

**Fig. 1:**
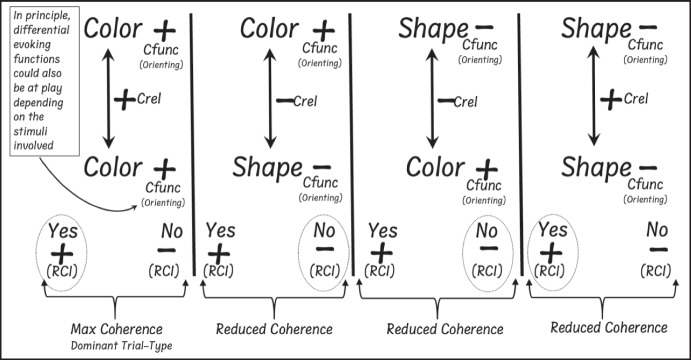
DAARRE Model Analysis of an IRAP Presenting Shape and Color Stimuli. *Note.* Circled response options indicate response deemed correct within history-consistent blocks of trials

The DAARRE model assumes that differential trial-type effects observed on the IRAP can be explained by the relative coherence between the Cfunc and Crel properties of the stimuli and response options employed across blocks of trials. In this context, response options such as “True” and “False” or “Yes” and “No” are termed relational coherence indicators (RCIs), given that they are often used to indicate coherence or incoherence between the label and target stimuli in a given IRAP (see Maloney & Barnes-Holmes, 2016, for a detailed treatment of RCIs). At the current time, three key sources of behavioral control are identified by the model: (1) the relation between the label and target stimuli (Crels); (2) the orienting and/or evoking functions of the label and target stimuli (Cfuncs); and (3) the coherence functions of the RCIs (e.g., “Yes” and “No”). The DAARRE model may thus explain differential trial-type effects based on the extent to which the Cfunc, Crel, and RCI properties cohere across blocks of trials. In the case of the larger color-color trial-type effect (relative to the shape–shape trial-type) mentioned above, it has been argued that participants tended to orient to the color more readily than to the shape stimuli based on the fact that color words occur far more frequently in natural language than shape words (see Finn et al., 2018, for further details). In conceptual terms, therefore, the color–color trial type consisted of relatively positive Cfunc and Crel properties, whereas for the shape–shape trial-type the Cfunc properties were less positive (see Figure 1). As such, there was maximal coherence among the Cfunc and Crel properties for the color–color trial-type, but reduced coherence for the shape–shape trial-type. This basic DAARRE model interpretation has received increasing support across a number of recent studies utilizing a range of stimuli with varying pre- and within-experimental behavioral histories (e.g., Bortoloti et al., 2019, 2020, 2023; Finn et al., 2019; Pidgeon et al., 2021; Pinto et al., 2020, Schmidt et al., 2021).

The important point at this stage is that research with the IRAP, particularly in the context of the DAARRE model, has highlighted that coherence among the functional properties of the stimuli appears to interact with their relational (Crel) properties in determining the patterns of responding observed on the IRAP (but see note 1 for a conceptual caveat). In noting this change in focus (i.e., function-to-relation), we are not suggesting that the original focus on relation-to-function was wrong, but simply that it needed to be supplemented with a clear recognition of the role of function-to-relation. The focus of the DAARRE model on the relative coherence among the Cfunc and Crel properties of the stimuli within an IRAP thus extends the analysis of the interaction between the relational and functional properties of stimuli. In conceptual terms, this new focus in the context of the DAARRE model seems to require a shift in emphasis within RFT itself, to which we now turn.

## Embracing a Field-Theoretic Approach within RFT

It is paradoxical that the shift in emphasis outlined above encourages a return to the earliest days of RFT when it was more focused on the analysis of complex relational networks involved in rule-governed behavior (see Hayes & Hayes, 1989), rather than as a theory of equivalence relations and the analysis of individual frames (see Hayes, 1991). Indeed, the original RFT formulation had a relatively strong field-like (Kantorian) influence, provided by L. J. Hayes (see Hayes et al., 2001, p. viii). What we will are suggesting in the current article is that the future of RFT appears to involve going back to its conceptual roots (see Barnes-Holmes et al., 2020, 2021, for related calls to a more field-based emphasis). That is to say that, at least for the current authors, the original Kantorian essence initially evident in RFT has reemerged in conceptualizing increasingly complex relational networks as involving a field of behavioral (verbal) interactants. By this we meant that, in our view, the individual elements within any given relational network do not exist independently of each other; rather they are actualized by their participation in a field of interactants. In Fig. 1, for example, the “+” orienting function for the label stimulus “Color” is defined, in part, relative to the “-” orienting function for the label stimulus “Shape.” The field of interactants that are actualized in the analysis of a specific IRAP performance thus provide the definition of a psychological event and the psychological event is the field—they are one and the same.

In calling for more field-based conceptual analyses in RFT, it may be useful to consider the DAARRE model in the context of Kantor’s formula for the psychological event. Let us begin by considering Kantor’s work in this area. According to Kantor (1958, p. 14), a psychological event is expressed via a formula that has been articulated recently in a text devoted to interbehaviorism by Hayes and Fryling (2023; see also Hayes & Fryling, 2018). In particular, the formula, PE = C (k, sf, rf, st, md, hi), is explained as follows:

- **PE** = The Psychological Event—This is another way of referring to the interbehavioral field or the “behavior segment.” The terms are used somewhat synonymously. This is a unit event of the subject matter of the science of psychology from an interbehavioral perspective.
- **C** = Indicates that the entire field of factors is one integrated whole.
- **k** = Specifies that no one psychological event can be identical to another event—as each is composed of a unique set and organization of factors.
- **sf** and **rf** = Stimulus function and response function. Important in this regard is that stimulus functions are distinguished from stimulus objects, and response functions are distinguished from responding organisms.
- **st** = **Setting factors** that are the immediate circumstances in the presence of which particular sf←→rf functions are taking place. Different sf←→rf functions occur in different settings.
- **md** = **Media of contact** refers to the means through which a biological organism contacts a physical stimulus object.
- **hi** =The **interbehavioral history** that represents the reactional biography and the evolution of stimulus functions (sf←→rf functions) throughout the organism’s history. (Hayes & Fryling, 2023, p. 50)

As suggested above, we believe that the DAARRE model may be usefully interpreted in terms of Kantor’s formulation of the psychological event. To appreciate this argument, we will explore how each of Kantor’s elements of the psychological event may be used to build an interbehavioral, field-based view of a DAARRE model analysis. In doing so, we should emphasize that the field-theoretical view we offer here is of the entire IRAP test performance and as such cannot collapse meaningfully into the analysis of individual IRAP responses (but see below). In other words, the psychological event in the current analysis is the entire IRAP performance and the entire IRAP performance is the psychological event. Thus, what we offer here is an intensely historical analysis, which includes the event consummating the completed IRAP test performance. Nevertheless, it is important to recognize that the functions of stimulating and responding on any given trial within an IRAP are momentary, with their previous instantiations becoming aspects of the setting in which new functions are actualized (see Hayes, 1992, for a relevant discussion of the psychological present). That is, the context of the IRAP test performance involves responding with respect to stimulating across each individual trial, such that the performance continues to occur with each new trial having a history missing in the previous trial. When viewed in this way, a more fine-grained analysis of responding on the IRAP could be conducted for other purposes.

### A Field-Based Interpretation of the DAARRE Model

It is important to note that Kantor asserts that the psychological event (PE) is identical to (=) the entire field of contributing factors (C). In this case the PE constitutes the overall IRAP performance to which the DAARRE model is applied (e.g., refer again to Fig. 1). The C element indicates that all of the factors contained within the DAARRE model should be considered as one integrated whole. That is, no one part of the model can be removed because doing so would fundamentally change the nature of the entire event. For example, if we removed the shape stimulus (and its “-” function), the “+” function of the color stimulus is by definition no longer part of the same integrated field event (because within the DAARRE model, the “+” and “-” functions are defined relative to each other). The k factor in the formula may be used to highlight that the DAARRE model is designed to capture a specific and unique psychological event. In other words, the model may be used to interpret any specific individual’s exposure to a particular IRAP. In this sense, if an individual was exposed to the same IRAP twice, each exposure may be considered a unique psychological event.

With respect to sf and rf elements, the DAARRE model clearly distinguishes between the stimulus objects and stimulus functions, and between response functions and responding individuals. For example, both color and shape may be interpreted as objects that “exist” independently of the DAARRE model, but their functions as “+” and “-” are only actualized within the model. Furthermore, the responses involved in picking “True” or “False” may be interpreted as the responses of individuals, but again, their response functions and how they do, or do not, cohere with the other elements within the DAARRE are only actualized within the model itself.

Next in Kantor’s formula is the setting factors (st) element. In the context of the DAARRE model, the functions of the elements within the IRAP will be defined in part by such factors. For example, it might be anticipated that conducting an IRAP in a relatively hot versus cold environmental setting may change the “+”/“-” functions of specific stimuli within an IRAP. Imagine, for instance, the stimuli were pictures of cold drinks versus open fires. In the hot setting, the cold drink stimuli may acquire “+” functions relative to “-” for the open fire stimuli, but the reverse might be the case in a cold setting. Indeed, some specific variables that have been manipulated in IRAP research to date may also be seen as important setting factors, such as the role of different types of instructions on IRAP performances (see Finn et al., 2018). Once again, the integrated nature of the psychological event or behavioral segment as reflected in the DAARRE model is highlighted in this example.^3^

Indeed, it is important to emphasize that setting factors extend well beyond a simple experimental manipulation, such as temperature. Setting factors can also involve, for instance, the wider cultural context in which an IRAP is delivered. For example, Power et al. (2017) compared IRAP performances using stimuli designed to assess racial biases among white Irish participants and Black African residents who had recently immigrated to Ireland. The white participants produced large differences among the trial-types that were interpreted at the time as indicative of racial bias. In contrast, the Black participants produced relatively small differences among the trial-types, suggesting the absence of racial bias. Perhaps the lack of differential trial-type effects for the latter group of participants was driven, at least in part, by the fact that they were completing the task in a nonnative language (i.e., English; see Bortoloti et al., 2023, for recent experimental support) and was perhaps also driven by completing the task for a white experimenter in a predominantly white country. In any case, the point is that setting factors in a field-based analysis can be broad in scope and invite increasingly expansive analyses of psychological events. It is also worth noting that it is important to distinguish between the terms and concepts we use in our detailed research (e.g., Crel and Cfunc properties) and the mainstream constructs (e.g., racial bias, culture) that are used to talk about the research and its implications for understanding our subject-matter. In doing so, we protect ourselves against falling into the mainstream psychological practice of treating behavior as a proxy for an ill-defined psychological construct, such as racial bias (e.g., see Fryling & Hayes, 2009; Kantor 1957; Smith, 2007).

It seems worth emphasizing the foregoing point that the interbehavioral field-based perspective appears to offer a strong prophylactic against the common practice of postulating reductionistic, mediating, and invariable hidden mentalistic (sometimes physiologically based) psychological constructs. When postulating such constructs, psychological science is immediately required to develop appropriate measures, often psychometric, that are notionally process pure or at least reasonably close. Within a field analysis, the development of such measures may be seen as a “fools errand” because each interbehavioral event is unique and the idea that there is some mentalistic psychological invariant that may be accessed via behavioral proxies is inconsistent with this view. As noted above, the interbehavioral field *is* the event and is dynamic and ever changing. Of course, analyzing any field event will involve invoking constructs but these are not mentalistic and are never confused with the event being analyzed. In the current context, analyzing IRAP patterns in terms of the constructs of Crel and Cfunc dominance, for example, does not involve an attempt to capture a mediating variable that determines behavior. Rather, these constructs are used in the analysis of the interbehavioral field that is actualized when a participant is exposed to an IRAP. In this sense the score on a particular trial-type on an IRAP is not a Crel or Cfunc property—it is the interaction between trial-types that is interpreted as relative dominance.

In moving to media of contact (md), it has to be acknowledged that virtually all IRAP studies to date have been conducted through visual stimulation and tactile responses. But of course, the DAARRE model could also apply to an IRAP that involved other media. In principle, for example, it would be possible to present an IRAP comprised of auditory stimulation and vocal response forms. Indeed, at the time of writing, we were aware of colleagues in Brazil that were attempting to develop an IRAP that presented stimuli in auditory rather than visual form. Of course, this example could be seen as relatively trivial in the grand scheme of field-based theorizing, but the example does serve to illustrate once again that IRAP research could be consistent with a more interbehavioral approach than has traditionally been the case.

Finally, the interbehavioral history (hi) is reflected in the DAARRE model in that the functions of the elements within the model are defined in part by the historical evolution of those functions. For example, in the context of the shapes and colors IRAP, the “+”/“-” functions for color and shape respectively were seen as evolving from the verbal histories of the experimental participants. As mentioned above, color words occur far more frequently in natural language than shape words, thus providing at least one historical basis for the dominance of the color–color over the shape–shape trial-type. In making this argument, we are not suggesting that this history *caused* the performance per se, but rather the DAARRE model *incorporates* that history itself. As such, the history cannot be separated from the model—it is one integrated whole. In this sense, the DAARRE should not be seen as a hypothetico-deductive model that specifies independent and dependent variables that interact in a linear and causal manner. Rather, the model simply provides a potential field of co-defining behavioral interactants that may be explored experimentally. Imagine, for example, a performance on a shapes-and-colors IRAP in which the shape-shape trial-type dominated over the color–color trial-type. In this case, the DAARRE model, if seen as an interbehaviorally defined psychological event, would suggest that a researcher seek to determine why the “+” and “-” functions were reversed across the shape and color stimuli (e.g., if the participant were an architect, shape stimuli may dominate in their history relative to colors). In other words, conceptualizing the DAARRE model as a model of a field of verbal interactants (i.e., interbehaviorally) readily invites conceptual and empirical questions about the histories that generate particular patterns within the field.

Of course, in making this argument we recognize that the preceding shapes and colors example may seem somewhat trivial. However, the importance of interbehavioral history in these analyses should not be underestimated. For example, even the immediate history preceding an experiment, including how an experimenter explains the study to a participant, are to be considered as interactants within the interbehavioral field. Indeed, the important role of experimental instructions, and previous experience with similar experimental procedures, has been reported in the IRAP literature (Finn et al., 2016, 2018). A focus on these types of historical variables certainly come to the fore when they are seen to be part-and-parcel of an IRAP performance participating in an interbehavioral field (in contrast to considering the IRAP as a proxy for a latent mentalistic psychological construct).

Having explained how the DAARRE model may be conceived of as capturing an interbehavioral psychological event, it seems important to elaborate on this view in terms of other related RFT concepts that have emerged recently in the literature. Consider that the DAARRE model, as applied to the shapes-and-colors IRAP, focused on orienting functions (i.e., participants oriented more readily to color than shape words). However, the DAARRE model also allows for other functions, namely evoking functions (i.e., ranging from aversive to neutral to appetitive; e.g., see Barnes-Holmes & Harte, 2022a). Furthermore, motivational contextual variables, although not generic response functions (similar to orienting and evoking), constitute a ubiquitous property of all psychological events that interact with relating, orienting and evoking, and thus motivational variables are part of the DAARRE model (see Gomes et al., 2020, for relevant empirical evidence for the role of motivational factors in IRAP performances). Each of these conceptual elements have been incorporated into a nonlinear, dynamical unit of analysis referred to as the ROE-M (*R*elating, *O*rienting, and *E*voking within a *M*otivational context; see Barnes-Holmes & Harte, 2022a). The ROE-M is conceptualized as an integrated unit of analysis for human psychological events and as such sits very well with the interbehavioral field-based psychological event as an integrated whole.

We recognize that the common meaning of the term “evoke” suggests a force of some sort; that is, a case of one thing making another thing happen. Related to this, although the term “to orient” does not have a forceful connotation, it seems to imply something that an organism does, and “to be oriented” seems to imply something a stimulus does. This apparent separation does not sit well with the interbehavioral idea that stimulating and responding are aspects of the same event. However, consistent with the argument made in Footnote 1, the “R” in the ROE-M cannot be separated from the orienting, evoking and motivating functions. As such, the patterns of stimulus-response functions, that emerge out of a human verbal/relational history, which we label orienting, evoking, and motivating, constitute the ROE-M as an integrated behavioral event. With that said, the functions of stimulating and responding that participate in a ROE-M emerge out of momentary events, but the unit itself should not be confused with those topographically separate events. The ROE-M in this sense is an interpretive construct, although as will be argued below, it may also be utilized for investigative purposes.

As an aside, it is important to note that “relating” within the ROE-M refers to AARR as defined within RFT, the details of which are beyond scope of current article. However, the reader is referred to Barnes-Holmes et al. (2020, 2021), where relating is described as being divided into different levels of relational complexity that can vary along different dimensions (coherence, complexity, derivation, and flexibility). Furthermore, the field concept has been invoked when conceptualizing relating in this way in the context of the ROE-M (Barnes-Holmes & Harte, 2022a), and we would argue is entirely consistent with the approach we are advocating for here.^4^

Before closing, it is also worth noting that the ROE-M has been described as a response unit contained within antecedent and consequential contextual variables, which may encourage relatively linear-based analyses, at least experimentally. In conceptual terms, however, the ROE-M is inherently nonlinear and the elements within it are co-defining. Thus, the ROE-M should be conceptualized as a unit of analysis that allows the analyst to construct the psychological event as a field of verbal interactants (i.e., contributing factors), as has been done with the DAARRE model above. As explained previously, the DAARRE model may be viewed readily through an interbehavioral/Kantorian lens. In effect, therefore, the concept of the ROE-M allows the analyst to generate potentially field-based analyses of the human psychological event.

In making the foregoing argument, we recognize that there may sometimes be a more linear focus on how antecedents and/or consequences affect the ROE-M (i.e., more in line with the traditional three-term contingency). Although such a linear approach may be helpful in the context of investigative or experimental research, it is important to emphasize that investigative constructs should not be conflated with the interpretive constructs of a purely field-based analysis. In effect, a more linear experimental focus appears to introduce some conceptual tension between a traditional contingency-based approach to the ROE-M versus a more field-based appreciation of the unit. However, clarity concerning the distinction between investigative and interpretive scientific language appears to resolve this tension, at least to some extent.

Indeed, recognizing the important distinction between interpretive and investigative constructs has allowed us to resolve the apparent conceptual conflict in our own research activities. In particular, on the one hand, we have used linear, contingency-based constructs in much of our research, and we cannot deny that this has been very productive and has helped us to achieve many of our analytic goals. On the other hand, in grappling with the complexities of human language and cognition, particularly within the context of research using the IRAP, the interbehavioral emphasis on not confusing investigative with interpretive constructs has proved invaluable. As such, we have argued that an IRAP performance may be usefully considered to be a dynamic and nonlinear field of verbal interactants. In short, linear-based analyses helped to produce the IRAP research program, and indeed RFT in general, but in doing so it has also brought us, at least in our view, into contact with the limitations of a purely linear-based approach. For example, we only appeared to make substantial progress in developing a functional-analytic account of IRAP performances when we began to cast those performances within a field-theoretic framework (i.e., the DAARRE model).

In closing, we fully acknowledge that much of what we have presented here may appear highly abstract and conceptual (although having emerged directly from our experimental research), and to some extent may be seen as potentially transforming RFT as widely understood, even within the RFT research community itself. However, in our defense, it should be recognized that RFT, or at least early work in the area, had a relatively strong Kantorian flavor, and indeed this was recognized in the seminal volume (Hayes et al., 2001, p. viii). It is perhaps ironic, therefore, that in struggling to develop RFT-based analyses of IRAP performances, some researchers (e.g., Barnes-Holmes & Harte, 2022b) were forced to return to one of the intellectual wells (the Kantorian tradition) from which RFT sprang forth. In particular, it seems essential to appreciate the impact of coherence *among* the functional properties of stimuli on patterns of relational responding, as captured, for example, by the DAARRE model. This alone seems to require an adjustment in the theory as widely understood, although we recognize that other developments have indeed occurred and are ongoing. Nevertheless, revisiting the original Kantorian essence within RFT seems to have facilitated new conceptual analyses within the theory in recent years. Indeed, if the interbehavioral approach is fully embraced, it would be transformative within the field of behavior-analysis in general, not just within RFT. In our view, the challenge involved in analyzing increasingly complex forms of human language and cognition presses us more and more towards field-based theorizing in some form or another. Although this move may be somewhat disorienting for the field (it certainly has been for us), we think it would be unwise not to more fully develop the interbehavioral approach within behavior-analysis. In any case, only time will tell whether or not this Kantorian move will be of benefit to the study of human language and cognition within the behavioral tradition, and perhaps beyond.

## Data Availability

This article does not contain any original data.

## Footnotes

1 RFT distinguishes between relational entailment and transformations of function because functions may transform in a large variety of patterns and it is scientifically useful to discriminate among these patterns in a relatively consistent way. However, it is fundamental that entailed relations within RFT are defined in terms of transformation of stimulus-response functions. For example, in the standard MTS performance described as symmetry or mutual entailment, the function of the sample from training transfers to the comparison and the function of the comparison from training transfers to the sample. As concluded by Dymond and Barnes (1994), “the observed pattern of a transfer of functions defines the entailed relations and thus the entailed relations . . . do not exist as a behavioral event until a specific transfer of functions has occurred (e.g., a transfer of matching-to-sample functions on a standard equivalence test)” (p. 264). In short, entailed relations *are* functions and strictly speaking, one does not have an impact upon the other in a mechanistic or causal sense, but rather relations and functions constitute complex patterns of stimulus–response interactions.

2 The IRAP was first conceptualized as a method for analyzing verbal relations as conceptualized within RFT, drawing as it did on earlier RFT work with the relational evaluation procedure (REP; see Hayes & Barnes, 1997). As such, the IRAP was designed to test previously established verbal relations (see Barnes-Holmes et al., 2008; see also Barnes-Holmes et al., 2006, for a brief history of the early development of the IRAP). However, the IRAP has been widely used from its earliest days as a measure of so-called implicit cognition (e.g., McKenna et al., 2007), which, it has been argued, detracted from its original focus on natural verbal relations (see Barnes-Holmes & Harte, 2022b). In recent research, though, the use of the method has become more closely aligned with its original purpose and, as we shall see, has served to stimulate a shift towards a more interbehavioral interpretation of its behavioral effects.

3 It is also important to recognize that the setting is not just a collection of factors in an event field, it is also their organization. Thus, a change in the setting (as when a factor is added or removed) rearranges the collection of factors—a new setting is therefore a new field.

4 When relating is conceptualized in a field-based manner, it may be useful to think of relating as evidence of the establishment/operation of substitute stimulation. For example, a relational response with respect to two arbitrary stimuli, such as A is more than B, may involve an interbehavioral history in which responding to formal or physical differences in stimuli is substituted with responding to stimuli that do not differ along the relevant formal dimension (i.e., treating A as if it *is* physically more than B). Likewise, it is important within a field-theoretic approach to conceptualize the orienting and evoking functions of stimuli, not as something stimuli do or cause, but rather as part of an ongoing behavioral interaction. For example, we can only understand the evoking property of a stimulus in the historical context in which that evoking property emerges in the wider interbehavioral field.
